# Post-COVID-19 Tourists’ Preferences, Attitudes and Travel Expectations: A Study in Guayaquil, Ecuador

**DOI:** 10.3390/ijerph19084822

**Published:** 2022-04-15

**Authors:** Miguel Orden-Mejía, Mauricio Carvache-Franco, Assumpció Huertas, Wilmer Carvache-Franco, Nathalie Landeta-Bejarano, Orly Carvache-Franco

**Affiliations:** 1 Facultat de Turisme i Geografia, Universitat Rovira i Virgili, 43480 Vila-seca, Spain; miguelangel.orden@estudiants.urv.cat; 2 Facultad de Turismo y Hotelería, Universidad Espíritu Santo, Samborondón 092301, Ecuador; mauricio2714@hotmail.com; 3 Department of Communication, Universitat Rovira i Virgili, 43002 Tarragona, Spain; sunsi.huertas@urv.cat; 4 Facultad de Ciencias Sociales y Humanísticas, Escuela Superior Politécnica del Litoral, Guayaquil 090615, Ecuador; 5 Carrera de Turismo, Universidad Técnica de Babahoyo, Babahoyo 120102, Ecuador; nlandeta@utb.edu.ec; 6 Facultad de Especialidades Empresariales, Universidad Católica de Santiago de Guayaquil, Guayaquil 090615, Ecuador; ocarvach@hotmail.com

**Keywords:** pandemic, crisis, destination choice, mental health, smart care, pricing strategy, safety, comfort, social distancing

## Abstract

Expectations about a destination influence the tourist experience during the travel process stages. In the post-COVID-19 normalcy, people are adjusting their priorities and social values. Therefore, it becomes crucial to identify tourists’ expectations before traveling. The objectives of this research were: (a) identify the preferences of tourists; (b) establish the attitudes of tourists; and (c) determine the expectations of tourists for post-COVID-19 destination selection. The study analyzed a sample of 491 people during pandemic lockdowns in Guayaquil, Ecuador. Statistical techniques such as exploratory and confirmatory factor analysis were used in data analysis. The results show that after the pandemic, tourists prefer urban tourism, followed by cultural tourism and traveling with relatives. It also shows a more responsible and supportive attitude when traveling. Likewise, the results support the dimensional structure that explains a set of post-pandemic tourist expectations. Five factors were identified: Smart Care, pricing strategy, safety, comfort, and social distancing. Finally, the theoretical and managerial implications of the results that will guide for tourism destination managers were discussed.

## 1. Introduction

The World Health Organization (WHO) reported in December 2019 the first case of coronavirus in Wuhan, China. As of 18 February 2020, the virus had caused more than 2200 deaths, and confirmed cases of COVID-19 infection exceeded 75,740 in the world [1]. On 31 January 2020, the WHO declared the coronavirus an international public health emergency, impacting the global economy, especially the tourism industry. From this perspective, the perception of COVID-19, travel risk and the willingness to change or cancel travel plans increased significantly during the pandemic due to the increase in confirmed cases worldwide [2]. Other reasons that influenced the slowdown in tourism were the susceptibility to COVID-19 infection, travel restrictions, and bans issued by governments [3]. Moreover, constant media coverage was the most influential factor in increasing risk perception [4].

The uncertainty about the future of the tourism industry forces us to rethink the different tourism management scenarios and analyze the impact of the pandemic on the emotional behavior of tourism demand. Hence, COVID-19 becomes a transformative opportunity [5] for researchers to explore, measure and predict the impacts of COVID-19 on tourism for monitoring and improving response strategies [6], especially in destinations where they have had a history of high COVID-19 incidences.

Guayaquil, Ecuador was chosen as the research subject in this study. Before the crisis, residents usually made tourism trips to domestic (especially coastal) and international destinations. Being a satellite city, there are various natural and cultural attractions in nearby areas. For this reason, residents practice certain forms of tourism such as: beach tourism, urban or city tourism, cultural tourism, ecotourism, and rural tourism.

Nonetheless, the impact of COVID-19 in Guayaquil was severe, significantly reducing tourist activity. The number of deaths during the coronavirus outbreak is among the worst in the world [7,8]. The global mortality ranking is headed by Guayas (including Guayaquil), according to the Financial Times, data on total deaths show that about 10,200 more people died during the months of March and April of the 2020 than in a typical year; i.e., an excess of deaths from the coronavirus of 485%, ranking with that figure as the city hardest hit by the coronavirus in the world [9].

On the other hand, academic literature on consumer behavior suggests that pre-purchase expectations determine product/service selection. In a post-pandemic context, it is necessary to deepen the analysis of the attitudes, behaviors and expectations of tourists before deciding to travel to predict future tourist demand and to be able to develop adequate recovery strategies. Therefore, understanding the new characteristics of tourists allows us to guide decision-making and choices of destinations behavior.

However, the current studies are focused mainly on the consequences of coronavirus on remodeling tourism, economic factors and resilience [10]. There are few studies based on tourist demand, especially consumer’s decision-making behavior [11]. Therefore, the objectives of this study are: (a) identify the preferences of tourists; (b) establish the attitudes of tourists; and (c) determine the expectations of tourists for post-COVID-19 destination selection.

The study was carried out in the city of Guayaquil, taking as a sample residents who in 2019 had made tourism and leisure trips, whether domestic or international.

The research questions posed by this study for the post-pandemic era are:

RQ1: What are the preferences of tourists?
RQ2: What are the attitudes of tourists on their trips?
RQ3: What are the expectations of tourists?

This work contributes to the emerging literature on the relationship between tourism and crisis [6]. Specifically, it provides exploratory data on tourists’ expectations towards a destination in a recovery phase. Moreover, the study provides destination management actors with valuable information for planning and managing internal tourism in a post-COVID-19 environment.

## 2. Literature Review

### 2.1. Tourism and COVID-19

The tourism industry has been one of the economic sectors most affected by the outbreak of the COVID-19 virus. The health crisis has caused the temporary closure of tourist services worldwide [12]. Several gastronomy-oriented and food companies have had to declare bankruptcy and closed their establishments completely. Likewise, concerts, (mega)events, festivals, and conferences were cancelled [13]. Most airlines were forced to reduce or cancel flights due to the coronavirus or government restrictions [14], as were hotels and tourist accommodation [15]. Based on the report of The United Nations World Tourism Organization [16], world tourism experienced an increase of 4% in 2021, compared to 2020 (415 million vs. 400 million). However, international tourist arrivals (overnight visitors) remained 72% below those of 2019, the year before the pandemic, according to preliminary UNWTO estimates. These are the figures that precede those of 2020, the worst year in the history of tourism when there was a 73% decrease in international arrivals. 

COVID-19 triggered an unprecedented crisis compared to other pandemics (Spanish flu of 1918, SARS, MERS, Ebola, or swine flu) or other crises in recent history, such as the terrorist attack of 11 September in the United States [17]. The COVID-19 virus, in a short period, generated enormous socio-cultural, political, and psychological impacts on various tourism actors, causing an unusual global crisis in our economic systems [6,18,19,20]. 

It is important to note that tourism plays an important role in public health [21], as well as the wellness components of vacations [22]. Therefore, financial support from governments towards the tourism and health sector is essential to ensure the balanced recovery of tourism. For example, the application of subsidies to promote the consumption of tourism, hotels and leisure, as well as subsidies to the health sector [23]. That is, recovery strategies must be holistic and innovative rather than direct [24]. Therefore, governments must increase the budget of the health and tourism sector to offer an adequate and affordable medical care service to its citizens and tourists. However, no policy or strategy works for all countries, because the impact and characteristics are unique in each territory. Mental health mechanisms include nature deprivation, family concerns, travel restrictions, and livelihood losses [25,26].

### 2.2. Tourist Destination and Types of Tourism

Tourist destinations (DT) are composed of various attributes that significantly affect tourists at different stages. Destination attributes are considered a group of dispersed elements that promote visitors to a destination [27]. In this sense, Ramón [28] reported that a tourist destination is a territorial system that integrates primary elements that make up its attractiveness and motivate the trip and secondary elements that facilitate consumption (accommodation, restaurants, and commerce).

A tourist destination is a package of tourist facilities and services that, like any other consumer product or service, comprises several multidimensional attributes that determine its attractiveness for a particular individual in a given situation [29].

Mayo and Jarvis [30] conceptualized destination attractiveness as related to the traveler decision-making process and traveler-specific benefits. Specifically, they defined destination attractiveness as a combination of the relative importance of individual benefits and the destination’s perceived ability to deliver these unique benefits. Logically, the more visitors believe that a tourist region could meet their vacation needs, the more attractive that destination region will be and the more likely they will select it as a potential travel destination.

Cultural tourism involves learning and experience as well as the consumption of tangible and intangible cultural attractions/products in a tourism destination. Ecotourism involves the observation, experience and appreciation of biological and cultural diversity. Rural tourism involves visitor’s experience related to products generally linked to agriculture, rural lifestyle/culture, angling and sightseeing. Urban/city destinations offer a broad and heterogeneous range of cultural, architectural, technological, social and natural experiences and products for leisure and business. Coastal tourism refers to recreational and sports activities that take place on the shore of a sea, lake or river [31].

### 2.3. Tourism Preferences

Tourist preferences are related to multiple travel attributes in terms of transport-accommodation consumption [32], price sensitivity [33], hotel and shopping choices [34], length of stay [35] and seasonality [36]. Vacation activity choices and preferences are an important aspect of tourist behavior. They influence tourists’ experiences, their levels of satisfaction, and their happiness with particular destinations [37]. People’s choices and preferences are shifting toward newer experiential and participatory activities that provide an escape from daily routines [38]. Therefore, activities at the destination are a crucial consideration in positioning and building destination brands [38]. 

For a tourist destination to fulfill its mission of attracting tourists, it is vital to recognize the needs of its potential visitors and discover the key elements that lead tourists to choose between one destination and another. Therefore, understanding tourists’ preferences and travel behavior is essential to develop infrastructure, products and services that satisfy their preferences [39]. In the context of risk, tourists make destination choices based on their individual perceptions of destination attributes, including risk-associated elements [40]. Likewise, income level is also a determining factor when selecting a destination, for example, in relation to the motives underlying the selection of destinations, low-income people were highly influenced by factors of “accessibility and discounts” despite the global health emergency [41]. For academics González-Reverté et al. [42], tourists with a previous environmental attitude are less interested in visiting mass tourism beach destinations in the future. For this reason, it is necessary to establish what preferences about tourist destinations exist in demand after the health crisis.

### 2.4. Tourist Attitude

Attitude significantly affects satisfaction [43]. Therefore, customer attitude is related to business performance [44]. Attitude generally refers to the number of customers/people (preferred/liked) or (liked/disliked) a particular object (e.g., product or service). It is usually demonstrated as a total evaluation of the objects, and it has been studied broadly in terms of behavior [45]. Attitude toward customer behavior refers to a “positive” or “negative” tendency to consistently react to certain behaviors, such as product use and product selection, according to research by Quintal et al. [46] In this sense, Ceylan et al. [47] and Untaru and Han [43] showed that consumer purchasing behavior has changed during the pandemic. The current scenario of COVID-19 expresses that the attitude of risk of an outbreak is a critical predictor of clients because the person realizes that entering a public place increases the probability of infection [48]. Previous economically oriented studies have shown how the COVID-19 crisis has revised dynamic customer reactions and consumption attitudes [47]. Therefore, in a study by Untaru and Han [43], in retail stores, customer attitudes towards protective measures have a solid mediating association with customer satisfaction and behavioral intentions, which increased satisfaction customer and return visit rate.

### 2.5. Tourist Expectations

Expectancy theory is based on various characteristics or attributes intended to be achieved or lead to a particular outcome [49]. In other words, expectations are preconceived and previously experienced perceptions of a product’s performance or attributes [50]. In this regard, Larsen [51] defines expectation as “an individual’s ability to anticipate, form beliefs and predict future events and states”.

Therefore, the tourist expectation is a “preconceived perception of the results of the trip” [52] built from various sources of information related to the tourist destination [53]; for example, tourism brochures, websites, and chatbots.

Furthermore, expectations are considered standards against which tourists assess a provider’s performance [54]. Therefore, the experience in a tourist destination is determined by the tourist’s expectations, the first element of the purchase decision. Thus, potential tourists’ expectations occur in the tourism industry before purchasing any tourism product. 

Heung and Quf [39] depicted these predictions as a set of attributes that describe a place as a travel destination (mental image), and Wang et al. [52] found that the cognitive/affective image of travelers shapes people’s expectations towards travel destinations. Significantly, satisfaction level, memories, choices, knowledge, and decisions respond to destination image [55]. Furthermore, a positive image of a destination formed by a synergy of destination attributes (e.g., tourism services and activities, infrastructure, attractions) influences decisions to choose a destination [56].

Several studies have explored tourists’ expectations. Tolls and Carr [57] analyzed the expectations of the tourist experience in a horseback riding center around notions of romance, nostalgia, relaxation, and escapism. In contrast, Larsen [51] argued that tourists’ expectations and in-trip perceptions and memories, shape the tourist experience and the basis for new preferences.

In tourist behavior, Hsu et al. [58] argued that expectation differently affects attitude, motivation, and loyalty towards a tourist destination [59]. Along these lines, Tsaur, Lin and Lin [60] found that the expectations of a memorable experience motivate visitors to participate in tourist activities. Thus, tourists’ expectations may become the reason for the trip.

## 3. Methodology

### 3.1. Study Area

**Figure 1:**
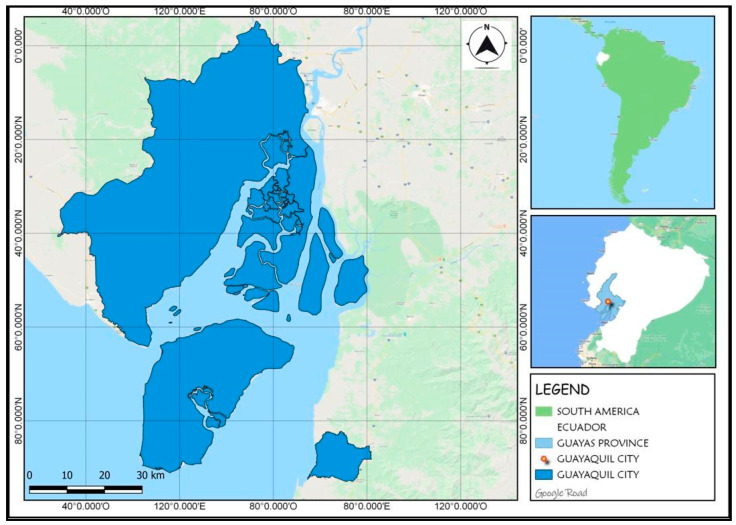
Guayaquil city, Ecuador.

Guayaquil is located on the coast of Ecuador in South America. It is a city with natural and cultural attractions visited by national and international tourists, confined at the time of COVID-2019. Guayaquil is the main economic city and one of the most populated, with approximately 2.7 million inhabitants [61], located at Latitude 2°11′41.30″ South and Longitude 79°52′55.77″ West (Figure 1). There are 142 registered accommodation establishments, 6352 rooms, 10,354 beds and with hotel capacity for 12,368 guests [62]. In this city, the Ministry of Public Health has 93 medical care sites, including health centers (first level), day and general hospitals (second level) and specialty hospitals (third level); while there are 217 private health establishments [63]. The main causes of death of Guayaquil residents in 2016 were ischemic heart disease (2116 cases) followed by Diabetes Mellitus (1376) (See Figure 1). 

Guayaquil is the capital of the Guayas province and offers a wide range of tourist attractions. Its shopping centers, parks, museums and boardwalks are the most visited by travelers who are enchanted by the magic that the so-called “Perla del Pacífico” offers. The Cathedral, the Seminary Park, the Governor’s Palace, the Santa Ana Hill, the Las Peñas neighborhood, the Simón Bolívar Malecón, the Samanes Park, the Guayaquil Historical Park, among others, are the destinations of tourist interest that day to day are visited by thousands of people who come to this cheerful and warm city.

Another alternative is tourism in the Gulf, a new fluvial alternative that articulates the main tourist sites around Guayaquil: Santay Island, Durán Train Station, Simón Bolívar Malecón and Samborondón Historical Park. In this area you can develop nature tourism (fauna and flora observation), active tourism (hiking, cycling), cultural and experiential activities, among others.

### 3.2. Survey Design

A questionnaire was designed to achieve the objectives. It included two sections: (1) the sociodemographic aspects of the respondents and their preferences for visiting a destination after the health emergency; (2) statements about tourists’ expectations for their next trip to a destination in recovery.

Since this was an exploratory study, an expert discussion elicited a total of 27 items organized into five factors (Smart Care, Pricing Strategy, Safety, Comfort, Social Distancing). All the items had a multiple-item measure, linked on a scale from 1 = strongly disagree to 5 = strongly agree: seven items for Smart Care, five items for pricing strategy, five items for safety, five for comfort, five for social distancing conducted an online pilot test (n = 25). Thus, the construction of the items was systematically examined to avoid ambiguous, vague, and unfamiliar terms [64]. After minor corrections and validation of the questionnaire, the final version was programmed into an online self-administered questionnaire to be completed by the respondents. 

### 3.3. Data Colletion

Guayaquil, the economic capital of Ecuador, was chosen as the research topic in this study. Residents of legal age who had traveled at least once (for leisure or vacation) in 2019 were selected for the sample. If they had not, their response was appreciated and the questionnaire was considered closed. Online surveys collected the data between April and May 2020 during the lockdown. The survey was designed in Google Forms and shared in Guayaquil using the social networks of Twitter, Facebook and Whatsapp. The sampling approach was non-probabilistic and applied convenience sampling to find errors and improve the survey. This non-random sampling technique was chosen due to its accessibility and the ease of reaching the respondent. When using this technique, habits, opinions, and points of view can be observed more easily.

Finally, the sample size was 491 valid responses for this study, and the infinite population was used, considering that there is no official number of tourists visiting the destination of Guayaquil. A ±5% margin of error, a confidence level of 95%, and a variance of 50% were used to obtain the most reliable results. 

### 3.4. Data Analysis

Descriptive statistics summarized the data. The SPSS 25 statistical software was used for data analysis:

1. A descriptive analysis was used to identify the participants’ profiles, preferences and attitudes towards a post-COVID-19 tourist destination.
2. An Exploratory Factor Analysis (EFA) was performed to facilitate the interpretation of the tourist expectation variables through a smaller number of variables or underlying factors.
3. Confirmatory factor analysis was applied to assess the adequacy of the measurements in terms of convergent and discriminant validity.

## 4. Results 

### 4.1. Study Simple Profile

Of the 491 responses, most participants were women, and one group was from the LGBT community. Most people were between 21 and 40 years old, 63.7% were single, and 83% had an undergraduate/postgraduate degree (See Table 1).

### 4.2. Preferences for Visits

Respondents would prefer urban tourism, followed by cultural tourism and rural tourism. In addition, most would like to visit a destination with their relatives. Likewise, they would be willing to travel with their partner and friends, and few would travel alone (see Table 2).

Results that answer our first research question in the post-pandemic era: What are the preferences of tourists on their next trips? Showing the results that they would prefer urban tourism, followed by cultural tourism and traveling with relatives. 

### 4.3. Travel Attitude

Fifty six percent of the participants mentioned that they would be more responsible and supportive when visiting their next destination in their tourist activities. Likewise, they preferred to visit less crowded destinations (51.2%) to avoid physical interaction with other tourists, thus guaranteeing an adequate distance.

Post-COVID-19 tourists will have a more respectful attitude towards the environment (41.2%). Thus, tourists will be aware of possible impacts on the destination. Potential tourists (39.2%) will be more careful with disinfection and hygiene in the tourist establishments of their destination (see Table 3).

Results that answer our second research question: RQ2: What are the attitudes of tourists on their trips? Evidencing the results that tourists would be more responsible and supportive when visiting their next destination in their tourist activities.

### 4.4. Exploratory Factor Analysis of Travel Expectations

This study was conducted using participants’ importance ratings regarding expectations of a post-COVID-19 destination. The Kaiser-Meyer-Olkin (KMO) measure of sample adequacy (MSA, Tokyo, Japan) was applied to determine the factorization of the data [65]. This paper found the KMO value of the data to be 0.916, indicating that it was excellent at sampling adequacy. Common method variance (CMV) bias was analyzed using Harman’s single factor test [66]. The results showed that the main factor explained 32.1% of the variance, below the 50% threshold, confirming that the bias is acceptable for data analysis. In addition, the Cronbach’s Alpha coefficient in the final scale of expectations of tourists reached a value of 0.930, which indicates a commendable internal consistency between the items of the scale.

The Bartlett sphericity test was also performed, since some variables have significant correlations [44]. In this case, the results of the Bartlett test indicate a level of significance (*p* ≤ 0.05). Hence, the data are suitable for EFA.

The maximum likelihood method with promax rotation was selected to identify the factor structure in the EFA application and obtain significant and interpretable factors because the object of study is the underlying causal structure of a given domain (expectations).

Data with a factor loading of less than 0.40 were not considered. The analysis was performed on 27 items, which explained 58.2% of the total variance and formed a structure of five dimensions with appropriate values: “Intelligent Care”, “Price Strategy”, “Safety”, “Comfort”, and “Social Distancing” (see Table 4).

According to Table 4, the first factor called “Smart Care” has the most significant explanatory power (35%) of the total variance. Thus, this factor is related to smart technologies such as chatbots for tour assistants, robots, applications, and artificial intelligence (AI).

The second factor was “Price strategy,” which reached 7.65% of the total variance related to low prices, discounts, and tourist services. For the third factor, “Security,” the results show that it comprised 6.79% of the total variance. This factor is related to protection and care issues contemplated in destinations once tourism is reactivated. The fourth factor, called “Comfort,” obtained 5.32% of the total variance. This factor is related to the intention to visit a destination where tourist activities can be carried out with small groups of people and where the itineraries are short.

In addition, tourists intend to visit a destination that ensures a perception of health and disinfection in tourist services. Therefore, tourists would be interested in service providers having COVID-19-free certification. The last factor, “Social distancing,” comprised 3.35% of the studied variance. This factor is related to social distancing in tourist services and activities and the tourist infrastructure of the destination, which is why they prefer to do tourism in open spaces and with fewer people.

**Figure 2:**
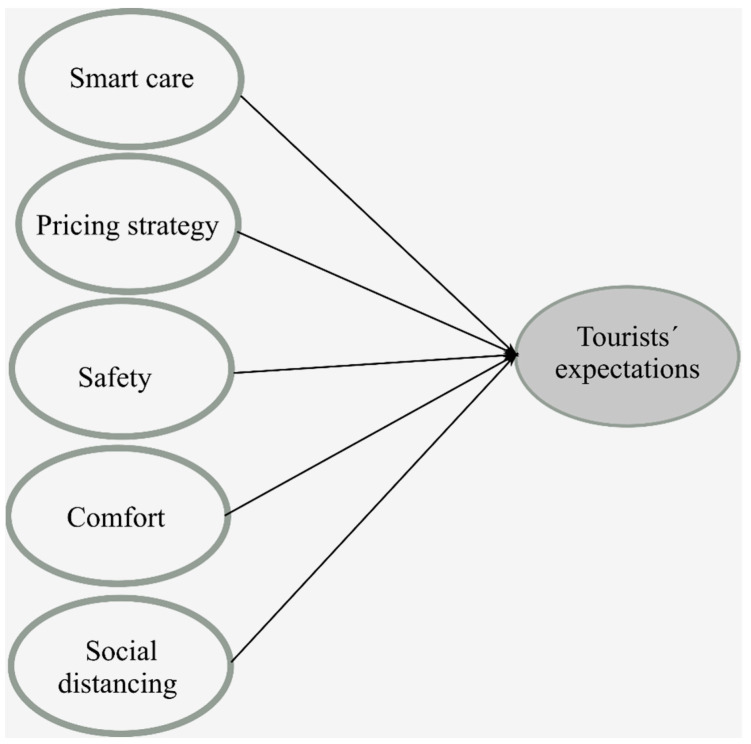
Tourists’ expectations for a destination in the post-COVID-19 recovery stage.

Five factors were revealed: Smart Care, Pricing Strategy, Safety, Comfort, and Social Distancing (see Figure 2).

Results that answer our third research question: RQ3: What are the expectations of tourists on their trips? It is evident that the main expectations of tourists are related to technological factors, biosecurity, and special-offer discounts.

### 4.5. Construct Validity

**Figure 3:**
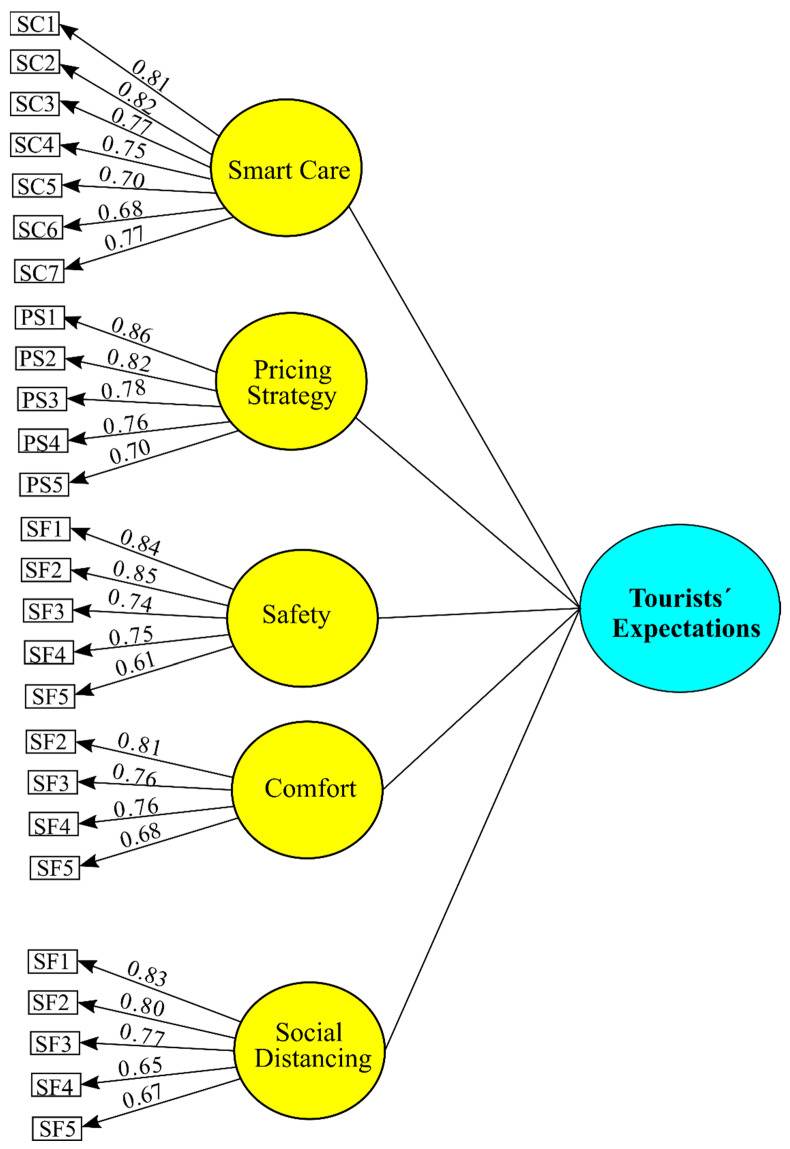
Confirmatory Factor Analysis (EFA). Maximum Likelihood Procedure.

Confirmatory factor analysis (CFA) was performed to test the psychometric properties of the measurement scales. The results confirmed the reliability and convergent validity of the measurement scales. In all cases, Cronbach’s Alpha and composite confidence are above the minimum required values of 0.7, and the AVE coefficients are above 0.5 [67]. Moreover, all items are significantly associated with their hypothetical factors at a 95% confidence level, and their standardized lambda coefficients are higher than 0.5 [68], confirming convergent validity. However, the item “Short itineraries (short-term tourist activities)” belonging to the comfort construct had to be eliminated, as it had a coefficient below the threshold (See Table 5 and Figure 3).

Finally, the results presented acceptable general adjustments ($x^{2}$ = 808.871; d*f* = 287; CMIN/d*f* = 2.818) at a level (*p* = 0.001). Goodness-of-fit indices were substantial (CFI = 0.929; TLI = 0.920; IFI = 0.930; RMSEA = 0.061) CFI, TLI and IFI values greater than 0.90 and an RMSEA value smaller than 0.08 are indicative of a good model fit [67].

The discriminant validity of the measurement scales was tested following the procedure proposed by [69], which compares the AVE coefficient for each pair of constructs with the estimated squared correlation between these two constructs. Thus, all constructs demonstrated acceptable discriminant validity because all intra-construct correlations were less than the square root of the AVE for each construct (see Table 6).

## 5. Discussion 

This study was carried out in the city of Guayaquil and was aimed at identifying the preferences, attitudes, and expectations of residents in planning their trips after the pandemic. The first objective was to identify the preferences of tourists on their trips. The results responding to RQ1 show that tourists would prefer urban tourism, followed by cultural tourism and rural tourism. Moreover, most of them would like to visit a destination with their relatives. The second objective of the present study was to establish the attitudes of tourists. Therefore, responding to RQ2, it has been identified that they would be more responsible and supportive when visiting their next destination in their tourist activities, as pointed out by Cameron and Shah [48] and Untaru and Han [43]. Likewise, they preferred to visit less crowded destinations to avoid physical interaction with other tourists, thus guaranteeing an adequate distance.

An interesting finding is that tourists prefer urban tourism in places that respect biosafety standards in less crowded places. It was also found that in coastal cities, tourists prefer urban tourism instead of going to the beaches, which are usually full of tourists [42].

As a third objective, the present study set out to determine the expectations of tourists for post-COVID-19 destination selection. In this way, the results responding to RQ show that the main expectations of tourists are related to technological factors, biosecurity and special offers and discounts. This study could significantly improve the visitor experience, compared to previous studies [51,57,59,60]. In this regard, Gretzel et al. [70] argued that information technology (IT) is the key to understanding the new conditions related to the pandemic on how we manage travel and our daily lives. All these are contributions to the academic literature on tourism in crisis, which until now has been very scarce. 

As practical implications, destination managers should create policies that encourage service providers to incorporate Smart Tourism Technologies (STT) such as chatbots, virtual assistants, biometrics (contactless), humanoid robots, augmented reality, AI, drones and sensors in tourist destinations. In addition, implementing these policies in the infrastructure of the destination can minimize physical contact, control social distancing and generate a positive image perception. In this study, chatbots or virtual tourist information assistants are the technologies that best explain the underlying structure of the smart care factor.

Through STT, destinations can (1) manage real-time destination tourism information and (2) achieve two-way communication between tourists and the local Destination Management Organization (DMO) and provide actionable information on destination conditions. 

Destination managers and private companies should implement customer pricing strategies, offering discounts on luxury services in hotels and restaurants and having special offers, due to the seasonality of the destination and because these variables will motivate visitors to take their travel decision, especially to a segment of tourists with less income [41].

Tourist destinations must invest in the expansion (in terms of breadth) of tourist infrastructure and recreation areas to guarantee the factor of social distancing. Likewise, it is essential to have social distancing signs that influence the behavior of tourists and residents. Additionally, it is necessary to generate prevention and distancing protocols in tourist services and activities to improve safety, hygiene and citizen transit.

In terms of safety, tourist destinations must form a management committee made up of representatives of the health, tourism, municipal and provincial secretariats to establish standards for monitoring and control of health protocols before, during and after the use of services and activities.

Tour agencies and operators must manage safe trips (health protocols), offering tour packages with previously certified services and ensuring that their providers implement the relevant health protocols to provide visitors with greater peace of mind during their stay at the destination.

Certified COVID-free destinations could meet the expectations of potential tourists. In addition, managers could implement training programs in sanitation and disinfection of tourist establishments and refresher courses in biosafety protocols to generate an environment under the sanitary requirements that post-COVID-19 tourists will demand.

## 6. Conclusions

The health system in Ecuador is universal and free. In Guayaquil, hospital care is offered on three levels and 24 h emergencies. For this, the city has modern accredited public hospitals that provide their services to both locals and tourists. There are also private clinics that serve tourists who have purchased international travel insurance.

The results of this research offer essential information. The EFA illustrates the dimensions of the expectations of tourists towards a destination in a recovery stage after COVID-19. This study provides exploratory information on the expectations of tourists about visiting a destination. Destination managers, destination and supply management organizations, and policy makers can benefit from this valuable information by identifying tourists’ expectations for choosing a destination in a post-COVID-19 scenario.

As theoretical implications, a good management of tourist expectations identified in this study could significantly improve the visitor experience, compared to previous studies. The main contribution of the study is the preferences, attitudes and expectations of the people who plan their visit and their travel after the pandemic. Therefore, tourists prefer urban tourism and travel with family members, and are more responsible and supportive. Five dimensions of expectations are evident: Smart Care, Pricing Strategy, Safety, Comfort, and Social Distancing. All of these findings imply a contribution to existing academic theory.

The study shows the temporality of the survey as the main limitation. Due to the confinement stage, the participants’ mental health might have been affected. Thus, the anxiety and trauma caused by the health emergency may have influenced the responses. Finally, future research is essential to address the term “COVID-19 phobia tourism”, which implies the tourist’s fear of contracting the COVID-19 virus during their next trip since it could be a determining factor for tourists when selecting a destination. Likewise, the tourist phobia due to COVID-19 generates new tourist behaviors, which will force destinations to redesign promotion strategies.

## Figures and Tables

**Table 1:** Demographic Profile.

Demographics	Categories	Frequency (n = 491)	%
Gender	Male	188	38.3
	Female	292	59.5
	LGBT	11	2.2
Age	>21 years old	119	24.2
	21–40 years old	313	63.7
	41–60 years old	54	11
	>61 years old	5	1.1
Education level	Primary education	2	0.4
	Secondary education	81	16.5
	University education	318	64.8
	Postgraduate degree	90	18.3
Marital status	Single	377	76.8
	Married	94	19.1
	Widower	1	0.2
	Divorced	19	3.9
Employment Status	Unemployed	35	7.1
	Self-employed	41	8.4
	Business owner	15	3.1
	Government employees	85	17.3
	Private employee	75	15.3
	Student	229	46.6
	Homemaker	10	2.0
	Retired	1	0.2

**Table 2:** Preferences.

Preferences	Frecuency	%
Type of tourism		
Urban or city tourism	242	49.3
Cultural tourism	82	16.7
Rural tourism	63	12.8
Sun and beach tourism	57	11.6
Ecotourism	47	9.6
Who would you make your trip with?		
With your family	263	53.6
With your partner	98	20.0
With friends	93	18.9
Alone	37	7.5
Others	-	-

**Table 3:** Attitude to travel to a destination.

Attitude	Frequency	%
More responsible and cautious	272	55.5
Search for less crowded places	251	51.2
Environmentally friendly	202	41.2
Overdone with cleanliness	192	39.2
Same as before, no change	36	7.3

**Table 4:** Exploratory Factor Analysis of Tourists’ expectation (n = 491).

Tourists’ Expectations and Associated Items	Loading	$h^{2}$
Smart Care: Eigenvalue = 9.89; Variance Explained = 35%		
SC1: Chatbot or virtual assistant for tourist information.	0.849	0.663
SC2: A.I. and local sensors to ensure management of crowds.	0.814	0.680
SC3: Mobility tracing apps.	0.758	0.593
SC4: Mobile application to identify COVID-19-free leisure services.	0.723	0.562
SC5: Biometrics (touchless) for identity control in leisure services.	0.720	0.507
SC6: Humanoid robots ultraviolet light for disinfection in leisure services.	0.697	0.472
SC7: App to know nearby medical offices, hospitals, and pharmacies.	0.680	0.576
Pricing Strategy: Eigenvalue = 2.47; Variance Explained = 7.65%		
PS1: Discount in leisure services offered by the destination.	0.943	0.783
PS2: Discounts on luxury leisure services.	0.833	0.676
PS3: Special offers in hotels, attractions and restaurants at the destination.	0.728	0.593
PS4: A value for money that guarantees an exclusive service.	0.711	0.581
PS5: Low prices in general for leisure services.	0.648	0.486
Safety: Eigenvalue = 2.19; Variance Explained = 6.79%		
SF1: Hygiene standards in tourist activities.	0.867	0.692
SF2: Biosecurity protocols in leisure services.	0.818	0.709
SF3: Hospital care following international criteria.	0.810	0.629
SF4: Detection and measurement of body temperature in tourism settings.	0.714	0.561
SF5: Medical insurance for hospital care.	0.662	0.457
Comfort: Eigenvalue = 1.82; Variance Explained = 5.32%		
CO1: Disinfection and sterilization of public spaces (smell of cleanliness).	0.937	0.728
CO2: Welcome host protocol that makes the visitor feel safe.	0.783	0.591
CO3: Small groups of people in tourist activities.	0.712	0.559
CO4: COVID-19-free certification in leisure services.	0.579	0.459
CO5: Short itineraries (short-term tourist activities).	0.390	0.298
Social Distancing: Eigenvalue = 1.30; Variance Explained = 3.35%		
SD1: Social distancing in leisure services.	0.980	0.782
SD2: Physical distancing in tourist activities.	0.787	0.645
SD3: Spaciousness in the tourist infrastructure (boardwalk, beach, trails, etc.)	0.715	0.573
SD4: Outdoor activities attractions.	0.499	0.420
SD5: Limited amount of co-presence of tourists in attractions and leisure service.	0.451	0.437
KMO = 0.916		
Chi squared = 613,471; *df* = 204; *p* < 0.001		
$\mathrm{Bartlett’}s\;\mathrm{Sphericity:}\;x^{2}$ = 7813,523; *df* = 351; *p* < 0.001		
Overall Cronbach’s Alpha (α): 0.930		

The $h^{2}$: value is the commonality of each. A 5-point Likert-type scale from 1 = strongly disagree to 5 = strongly agree.

**Table 5:** Scale Items and Confirmatory Factor Analysis result (n = 491).

Factor	Variables	Stand. Coef.	Cronbach’s Alpha	Composite Reliability	AVE	Mean (SD) ^a^
Smart Care			0.901	0.901	0.567	
	SC1	0.807				4.12 (0.867)
	SC2	0.818				4.25 (0.847)
	SC3	0.780				4.23 (0.800)
	SC4	0.746				4.25 (0.844)
	SC5	0.676				3.94 (0.926)
	SC6	0.655				4.03 (0.909)
	SC7	0.773				4.42 (0.742)
Pricing Strategy			0.886	0.888	0.615	
	PS1	0.861				3.97 (1.07)
	PS2	0.818				3.93 (1.13)
	PS3	0.776				4.15 (1.01)
	PS4	0.762				4.35 (0.966)
	PS5	0.699				4.25 (0.940)
Safety			0.875	0.875	0.586	
	SF1	0.841				4.76 (0.590)
	SF2	0.854				4.64 (0.675)
	SF3	0.743				4.59 (0.704)
	SF4	0.752				4.65 (0.720)
	SF5	662				4.49 (0.811)
Comfort			0.818	0.840	0.568	
	CO1	0.805				4.57 (0.718)
	CO2	0.762				4.55 (0.761)
	CO3	0.757				4.49 (0.799)
	CO4	0.685				4.50 (0.824)
Social Distancing			0.854	0.864	0.561	
	SD1	0.835				4.47 (0.737)
	SD2	0.797				4.46 (0.739)
	SD3	0.768				4.50 (0.741)
	SD4	0.654				4.64 (0.637)
	SD5	0.674				4.28 (0.916)

^a^ SD = Standard Deviation.

**Table 6:** Result of discriminant validity (Fornell-Larcker).

Construct	1	2	3	4	5
1. Smart Care	**0.753**				
2. Pricing strategy	0.484 ***	**0.784**			
3. Safety	0.382 ***	0.331 ***	**0.766**		
4. Comfort	0.564 ***	0.424 ***	0.415 ***	**0.753**	
5. Social distancing	0.605 ***	0.577 ***	0.485 ***	0.625 ***	**0.749**

Note: *** *p* ˂ 0.001; The square root of AVEs are shown diagonally in bold.

## Data Availability

Not applicable.
